# LncRNA FAM181A-AS1 promotes gliomagenesis by sponging miR-129-5p and upregulating ZRANB2

**DOI:** 10.18632/aging.103391

**Published:** 2020-10-20

**Authors:** Xin Jiang, Dong Chen

**Affiliations:** 1 Department of Orthopedics, China-Japan Friendship Hospital, Beijing, China

**Keywords:** FAM181A-AS1, glioma, cell proliferation, miR-129-5p

## Abstract

In this study, we investigated the functional and clinical significance of the long non-coding RNA (lncRNA) *FAM181A-AS1* in human gliomas. TCGA, GTEx and CGGA database analyses showed that high *FAM181A-AS1* expression correlates with advanced tumor stage and poor survival of glioma patients. *FAM181A-AS1* expression is higher in glioma cell lines compared to normal human astrocytes (NHA). CCK-8, EdU, and colony formation assays show that *FAM181A-AS1* knockdown decreases proliferation and colony formation in glioma cells, whereas, *FAM181A-AS1* overexpression reverses these effects. Bioinformatics analysis showed that miR-129-5p is a potential target of *FAM181A-AS1.* MiR-129-5p expression negatively correlates with *FAM181A-AS1* expression in glioma patients. Dual luciferase reporter assays confirmed that miR-129-5p binds directly to *FAM181A-AS1* in glioma cells. RNA immunoprecipitation (RIP) assays using anti-Ago2 antibody pulled down *FAM181A-AS1* with miR-129-5p. Bioinformatics analysis identified *ZRANB2* as a potential miR-129-5p target gene. Dual luciferase reporter assays confirmed that miR-129-5p binds directly to the 3’-UTR of *ZRANB*2 mRNA. Furthermore, miR-129-5p overexpression or *ZRANB2* knockdown reduces proliferation and colony formation of *FAM181A-AS1* overexpressing glioma cells. These findings show that *FAM181A-AS1* promotes gliomagenesis by enhancing ZRANB2 expression by sponging of miR-129-5p.

## INTRODUCTION

Glioma accounts for 46-70% of all patients with central nervous system (CNS)-related cancers [1]. The other CNS-related cancers include oligodendrogliomas, astrocytomas, glioblastomas, and ependymomas [2]. Although chemo-radiotherapy and surgical resection techniques have greatly improved in recent decades, the prognosis of glioma patients remains highly unfavorable [3, 4]. Therefore, identifying molecular mechanisms that regulate gliomagenesis is pivotal to improvements in glioma therapy.

Mutations in isocitrate dehydrogenase (IDH) profoundly affect growth and progression of gliomas as well as therapeutic outcomes [5]. Several IDH mutations are associated with increased survival of patients with glioma [6, 7].

Long noncoding RNAs (lncRNAs) are a class of RNAs that are >200 nucleotides in length and do not contain any open reading frames (ORFs) that can be translated into proteins [8, 9]. They account for more than 80% of ncRNAs and are less conserved than the microRNAs [10]. Moreover, lncRNAs regulate cellular growth, survival, and differentiation, genomic imprinting, epigenetic and post-transcriptional regulation of gene expression, alternate splicing, chromatin modifications, subcellular transport, and inflammatory mechanisms [11–14]. Dysregulation of lncRNAs is associated with the growth and metastasis of several tumors including gliomas [15–17]. The role of lncRNA FAM181A-AS1 in gliomas is not known. Therefore, in this study, we investigated the function and clinical significance of FAM181A-AS1 in gliomas.

## RESULTS

### *FAM181A-AS1* expression is significantly higher in human glioma tissues

**Figure 1:**
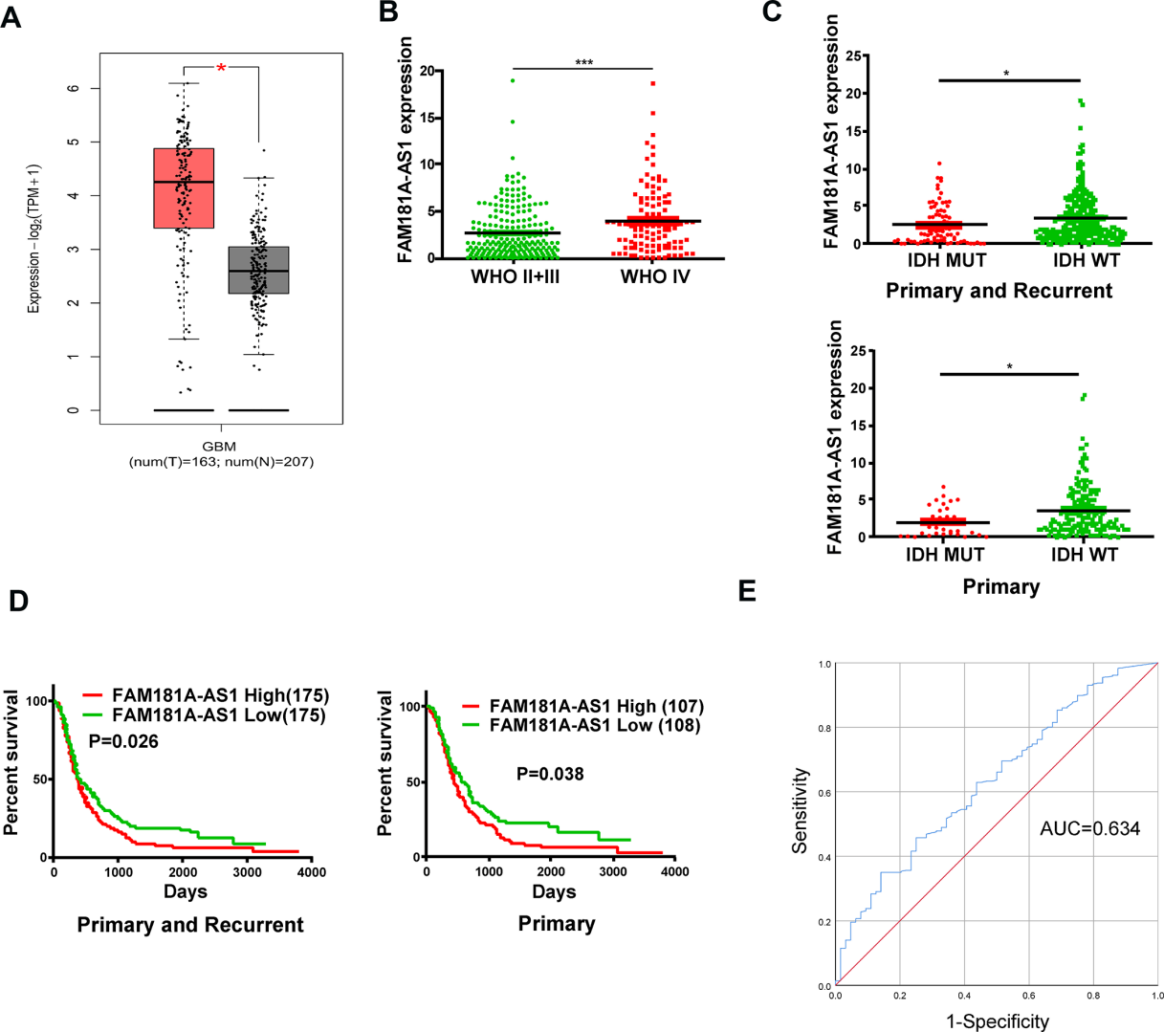
**High lncRNA *FAM181A-AS1* expression correlates with poor prognosis of glioma patients.** (**A**) TCGA and GTEx database analyses show *FAM181A-AS1* expression in glioma (n=163) and non-cancerous brain tissue samples (n=207). (**B**) CGGA database analysis shows *FAM181A-AS1* expression in WHO stage II/III (n=105) and WHO stage IV (n=109) glioma patients. (**C**) CGGA database analysis shows *FAM181A-AS1* expression in glioma patients with wild-type (n=175) or mutant IDH genotypes (n=33). (**D**) Kaplan-Meier survival curve analysis shows overall survival of glioma patients with high or low *FAM181A-AS1* expression. (**E**) ROC curves analysis shows the sensitivity and specificity of *FAM181A-AS1* expression in glioma samples. Note: *P<0.05, ***P<0.001; data are represented as means ± SD.

We analyzed RNA-seq data in the GTEx and TCGA databases and found that lncRNA *FAM181A-AS1* expression was significantly higher in human glioma samples (n=163) compared to the corresponding controls (n=207, Figure 1A). Moreover, analysis of the CGGA database showed that *FAM181A-AS1* expression was significantly higher in patients with WHO stage IV glioma compared to those with WHO stage II/III glioma (Figure 1B).

### High *FAM181A-AS1* expression correlates with unfavorable prognosis in glioma patients

Next, we analyzed the clinical significance of *FAM181A-AS1* expression in glioma patients. Previous studies show that glioma patients with IDH mutations are associated with increased survival rates [18, 19]. The CGGA database analysis showed that *FAM181A-AS1* expression was significantly reduced in glioma patients harboring *IDH* mutations compared to those without *IDH* mutations (Figure 1C). Furthermore, Kaplan-Meier survival curve analysis demonstrated that survival rates of glioma patients with high *FAM181A-AS1* expression was significantly lower than those with low *FAM181A-* *AS1* expression (Figure 1D). Subsequently, receiver *AS1* expression in glioma patients was 0.634 (Figure 1E). These data demonstrate that *FAM181A-AS1* is a potential prognostic biomarker for glioma patients.

### *FAM181A-AS1* downregulation inhibits cell proliferation and promotes cell apoptosis

**Figure 2:**
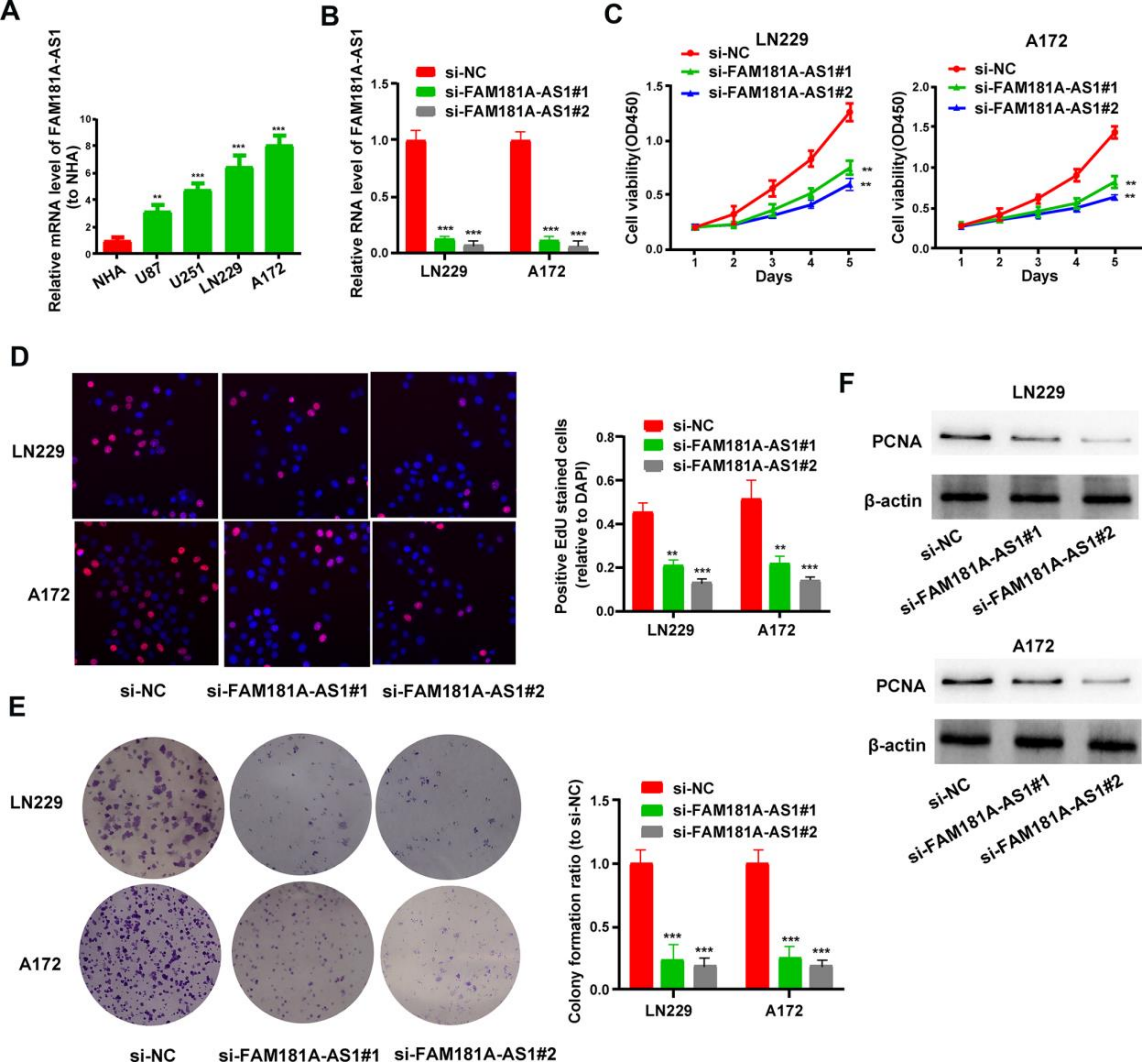
***FAM181A-AS1* knockdown inhibits glioma cell proliferation and colony formation.** (**A**) QRT-PCR analysis shows *FAM181A-AS1* levels in the four glioma cell lines, namely, LN229, A172, U87 and U251, and the control normal human astrocyte (NHA) cell line. (**B**) QRT-PCR analysis shows *FAM181A-AS1* levels in si-NC- and si-*FAM181A-AS1*-transfected LN229 and A172 cell lines. (**C**, **D**) CCK-8 and EdU assay results show proliferation of si-NC- and si-*FAM181A-AS1*-transfected LN229 and A172 cell lines. (**E**) Colony formation assay results show the numbers of colonies formed by si-NC- and si-*FAM181A-AS1*-transfected LN229 and A172 cells. (**F**) Western blot analysis shows the levels of PCNA protein in si-NC- and si-*FAM181A-AS1*-transfected LN229 and A172 cell lines. Note: **P<0.01, ***P<0.001; data are represented as means ± SD.

Next, we analyzed the role of *FAM181A-AS1* in the growth and proliferation of glioma cell lines. QRT-PCR analysis showed that *FAM181A-AS1* expression was significantly higher in four glioma cell lines, U87, U251, LN229 and A172, compared to the normal human astrocyte (NHA) cell line (Figure 2A). Comparatively, *FAM181A-AS1* expression was significantly lower in U87 and U251 cell lines compared to LN229 and A172 cell lines. Hence, we performed *FAM181A-AS1* knockdown in LN229 and A172 cell lines by transfecting them with siRNA against *FAM181A-AS1*. *FAM181A-AS1* levels were significantly reduced in si-*FAM181A-AS1*-transfected LN229 and A172 cells compared to si-NC-transfected LN229 and A172 cells (Figure 2B). CCK-8, EdU and showed significantly reduced proliferation and colony formation capacity (Figure 2C–2E) compared to si-NC-transfected LN229 and A172 cells. Western blot results showed that the levels of proliferation-related protein, PCNA, were significantly reduced in the si-*FAM181A-AS1*-transfected LN229 and A172 cells compared to the si-NC-transfected LN229 and A172 cells (Figure 2F). Furthermore, we analyzed the effects of *FAM181A-AS1* knockdown on the status of cell cycle progression and apoptosis of LN229 and A172 cells by flow cytometry. The results showed that *FAM181A-AS1* knockdown significantly reduced the percentage of S-phase cells, suggesting G0/G1 phase cell cycle arrest (Supplementary Figure 1A). Moreover, *FAM181A-AS1* knockdown LN229 and A172 cells showed increased apoptosis compared to controls (Supplementary Figure 1B). Furthermore, western blot analysis showed that the levels of cyclin D1 (cell cycle regulating protein) and Bcl-2 (anti-apoptotic or pro-survival protein) were significantly reduced in the si-*FAM181A-AS1*-transfected LN229 and A172 cells compared to the si-NC-transfected LN229 and A172 cells (Supplementary Figure 1C).

### *FAM181A-AS1* overexpression promotes cell proliferation and colony formation in glioma cell lines

**Figure 3:**
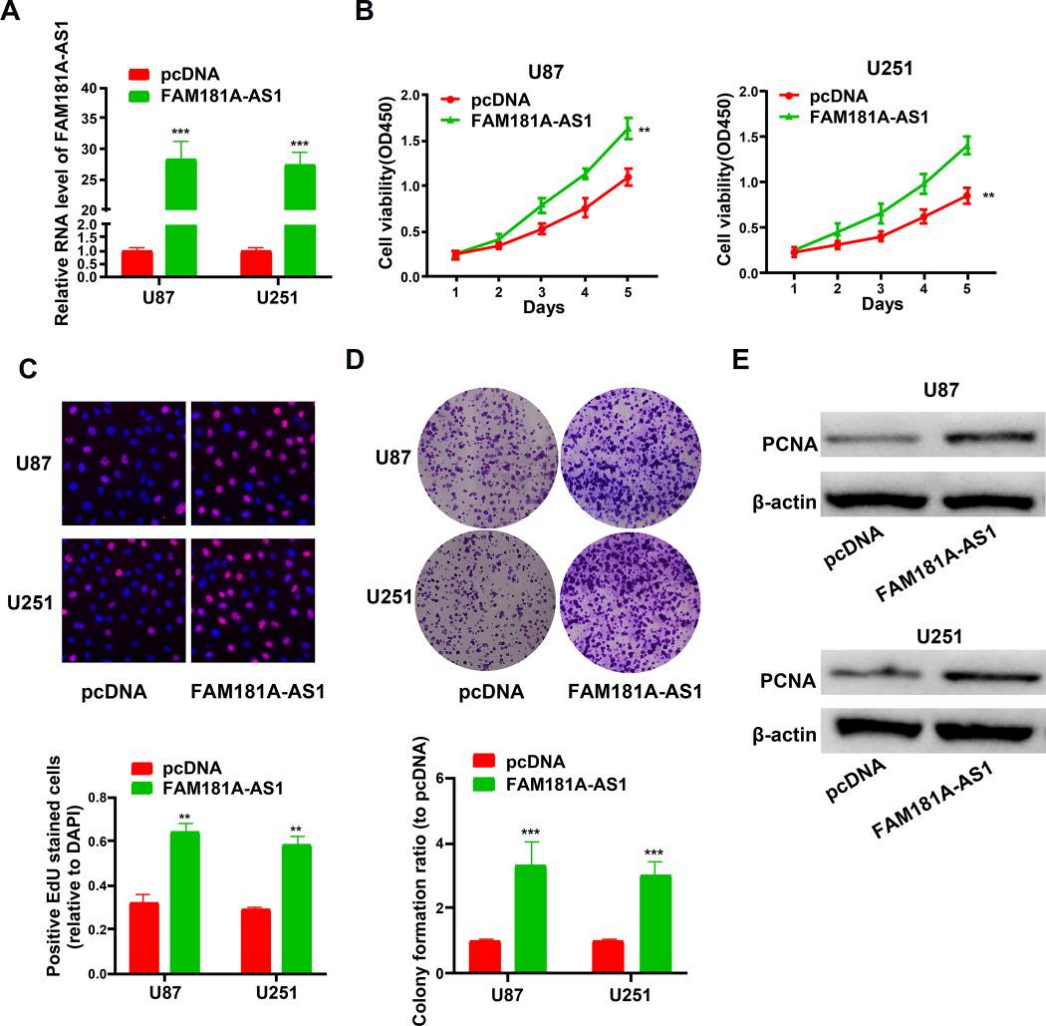
***FAM181A-AS1* overexpression promotes cell proliferation and colony formation.** (**A**) QRT-PCR analysis shows *FAM181A-AS1* levels in pcDNA3.1- and pcDNA3.1-*FAM181A-AS1-*transfected U87 and U251 cell lines. (**B**, **C**) CCK-8 and EdU assay results show proliferation of control and *FAM181A-AS1-*overexpressing U87 and U251 cell lines. (**D**) Colony formation assay shows the total numbers of colonies formed by control and *FAM181A-AS1*-overexpressing U87 and U251 cell lines. (**E**) Western blot analysis shows the levels of PCNA protein in control and *FAM181A-AS1*-overexpressing U87 and U251 cell lines. Note: **P<0.01, ***P<0.001; data are represented as means ± SD.

Next, we analyzed the effects of *FAM181A-AS1* overexpression on the growth and proliferation of U87 and U251 cell lines. QRT-PCR analysis showed that *FAM181A-AS1* levels were significantly higher in *FAM181A-AS1*-overexpressing U87 and U251 cells compared to the controls (Figure 3A). Furthermore, CCK-8, EdU and colony formation assays showed significantly higher proliferation (Figure 3B, 3C) and colony formation capacity (Figure 3D) in the *FAM181A-AS1*-overexpressing U87 and U251 cells compared to the controls. Moreover, western blot analysis showed that PCNA protein levels were significantly higher in the *FAM181A-AS1*-overexpressing U87 and U251 cells compared to the controls (Figure 3E).

### Low miR-129-5p expression correlates with advanced tumor stage and unfavorable survival of glioma patients

**Figure 4:**
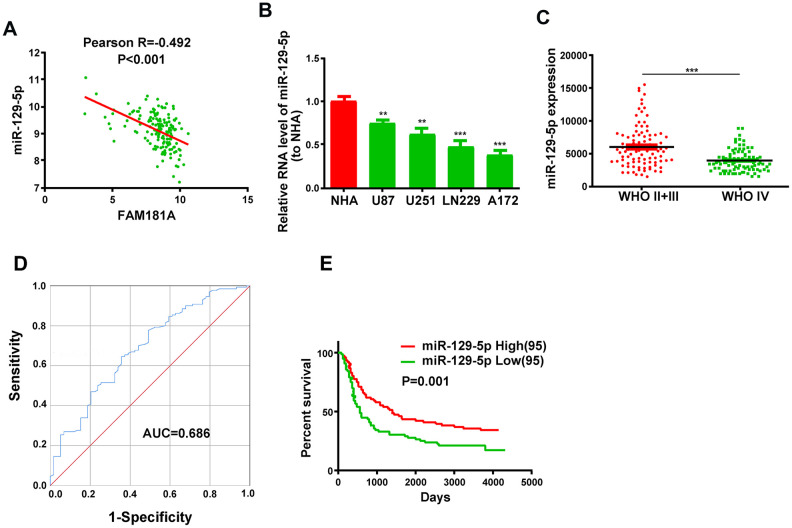
**Low miR-129-5p expression correlates with advanced tumor stage and poor survival of glioma patients.** (**A**) Pearson correlation analysis shows the relationship between *FAM181A-AS1* and miR-129-5p expression in glioma patients from the CGGA database. (**B**) QRT-PCR analysis shows miR-129-5p expression in the four glioma cell lines and the control NHA cell line. (**C**) QRT-PCR analysis shows miR-129-5p expression in patients with WHO stage II/III or WHO stage IV glioma from the CGGA database. (**D**) ROC curve analysis shows the sensitivity and specificity and AUC value of miR-129-5p expression in glioma samples. (**E**) Kaplan-Meier survival curve analysis shows the overall survival of glioma patients with high or low miR-129-5p expression. Note: **P<0.01, ***P<0.001; data are represented as means ± SD.

Next, we analyzed the microRNA.org database [20] to determine *FAM181A-AS1*-binding microRNAs and identified miR-129-5p as a potential target. The CGGA database analysis showed that miR-129-5p expression negatively correlated with *FAM181A-AS1* expression in glioma patients (Figure 4A). Moreover, miR-129-5p expression was significantly lower in the glioma cell lines compared to the NHA cell line (Figure 4B). Besides, miR-129-5p expression was significantly lower in the patients with WHO stage IV glioma compared to those with WHO stage II/III glioma (Figure 4C). ROC curve analysis showed that the area under curve (AUC) value for miR-129-5p was 0.686 (Figure 4D). Kaplan-Meier survival curve analysis showed that the survival of glioma patients with low miR-129-5p expression was significantly shorter compared to those with high miR-129-5p expression (Figure 4E).

### *FAM181A-AS1* directly binds to miR-129-5p in the glioma cells

**Figure 5:**
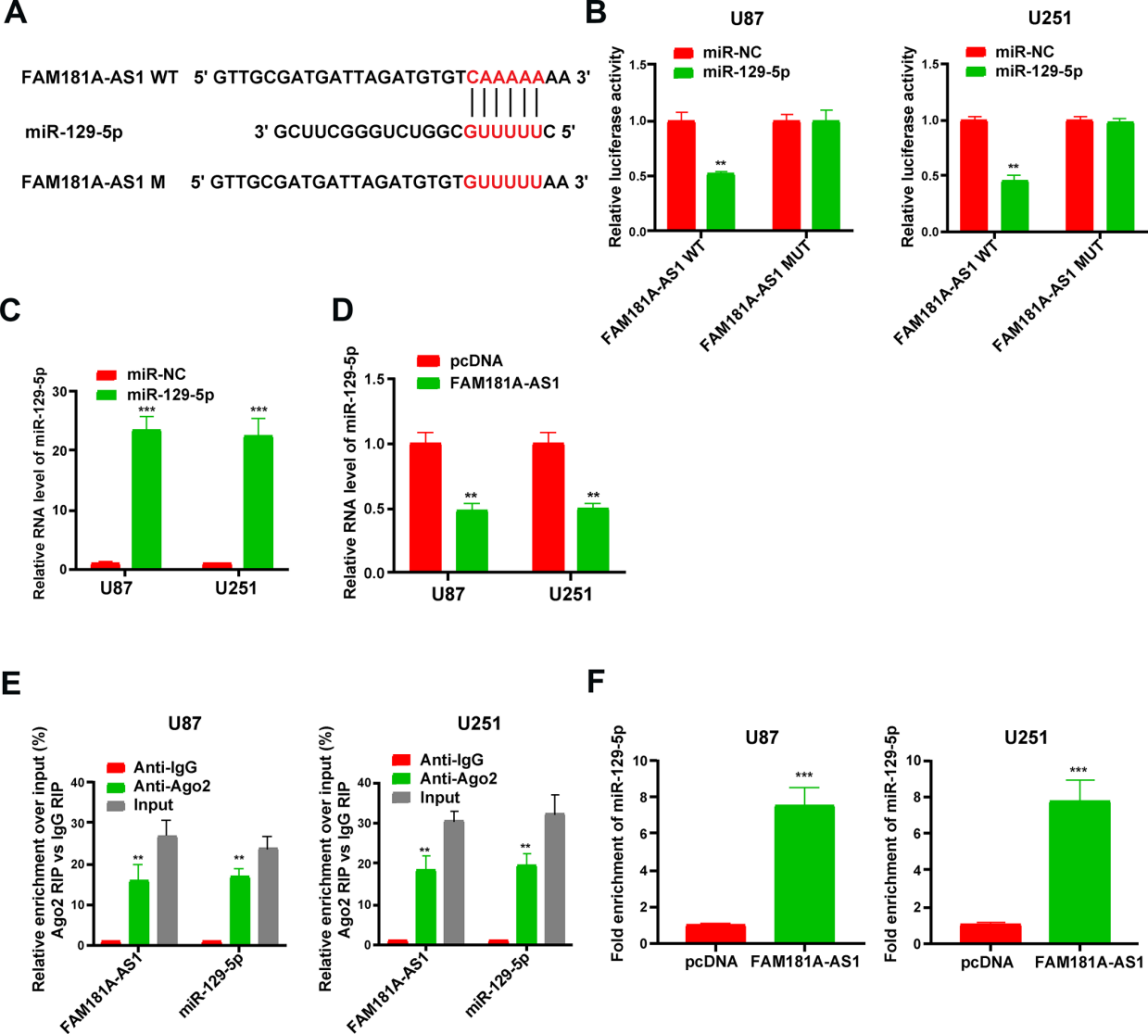
**LncRNA *FAM181A-AS1* sponges miR-129-5p in glioma cells.** (**A**) The diagrammatic representation shows miR-129-5p-binding sites in the wild-type and mutant *FAM181A-AS1*sequences. (**B**) Dual luciferase reporter assay results show relative firefly luciferase activity in U87 and U251 cell lines transfected with miR-129-5p mimic and vectors containing *FAM181A-AS1* with wild-type (WT) or mutant (MUT) miR-129-5p-binding sites. (**C**) QRT-PCR analysis shows miR-129-5p levels in control and miR-129-5p mimic-transfected U87 and U251 cell lines. (**D**) QRT-PCR analysis shows miR-129-5p levels in control and *FAM181A-AS1* overexpressing U87 and U251 cell lines. (**E**) Ago2 RIP assay results show enrichment of *FAM181A-AS1* and miR-129-5p using qRT-PCR in Ago2 protein pulldown samples from U87 and U251 cell lines using anti-Ago2 antibody. (**F**) QRT-PCR assay shows the levels of miR-129-5p in the RNA-RNA complexes pulled down using the *FAM181A-AS1* biotinylated probe in comparison with the negative control. Note: **P<0.01, ***P<0.001; data are represented as means ± SD.

Since lncRNAs function as competitive endogenous RNAs (ceRNAs) and function by sponging miRNAs, we explored the effects of *FAM181A-AS1* overexpression or knockdown on the levels of miR-129-5p. Figure 5A shows the miR-129-5p binding sites in the wild-type and mutated *FAM181A-AS1*. Dual luciferase reporter assay results showed that luciferase activity of U87 and U251 cells transfected with miR-129-5p mimic plus vector containing wild-type *FAM181A-AS1* was significantly lower than those transfected with miR-129-5p mimic plus mutant *FAM181A-AS1* (Figure 5B). Moreover, *FAM181A-AS1* overexpression in the U87 and U251 cells suppressed the luciferase activity of wild-type miR-129-5p, but did not affect the luciferase activity of mutant miR-129-5p MUT (Supplementary Figure 2A, 2B). QRT-PCR analysis showed that miR-129-5p levels were significantly higher in glioma cell lines transfected with the miR-129-5p mimic compared to the corresponding controls (Figure 5C). Furthermore, miR-129-5p expression was significantly reduced in pcDNA3.1-*FAM181A-AS1*-transfected glioma cell lines compared to those transfected with the control pcDNA3.1 vector alone (Figure 5D). RIP assay analysis using the anti-Ago2 antibody showed that both *FAM181A-AS1* and miR-129-5p were pulled down together compared to the negative control (Figure 5E). Moreover, qRT-PCR analysis also showed that miR-129-5p was enriched in the RNA-RNA complexes pulled down by the *FAM181A-AS1* biotinylated probe (Figure 5F). These data confirm that *FAM181A-AS1* interacts directly with miR-129-5p.

### MiR-129-5p overexpression inhibits proliferation of *FAM181A-AS1*-overexpressing glioma cells

**Figure 6:**
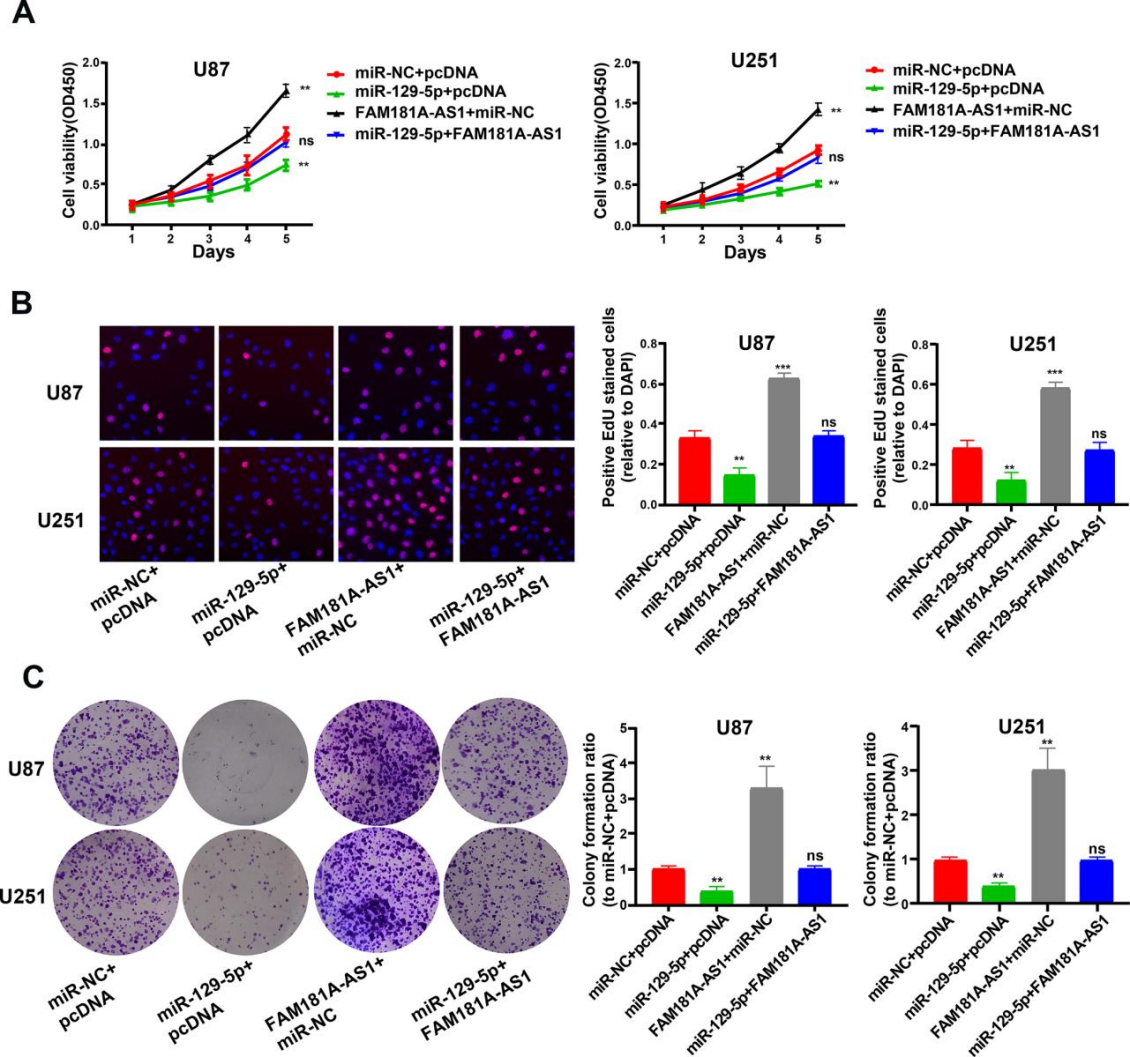
**MiR-129-5p mimic treatment reduces cell proliferation and colony formation *FAM181A-AS1-*overexpressing glioma cell lines.** (**A**, **B**) CCK-8 and EdU assay results show proliferation of *FAM181A-AS1*-overexpressing U87 and U251 cell lines transfected with miR-NC (negative control) or miR-129-5p mimic. (**C**) Colony formation assay results show the total numbers of colonies formed by *FAM181A-AS1*-overexpressing U87 and U251 cell lines transfected with miR-NC (negative control) or miR-129-5p mimic. Note: **P<0.01, ***P<0.001; ns, not statistically significant; data are represented as means ± SD.

Next, we assessed the functional relationship between *FAM181A-AS1* and miR-129-5p expression in glioma cell lines by co-transfecting U87 and U251 cells using miR-129-5p mimic and the *FAM181A-AS1* overexpression vector. CCK-8, EdU, and colony formation assays showed that miR-129-5p mimic reduced *FAM181A-AS1-*dependent proliferation and colony formation capacity in glioma cells (Figure 6A–6C).

### MiR-129-5p specifically binds to the 3’UTR of *ZRANB2* in glioma cells

**Figure 7:**
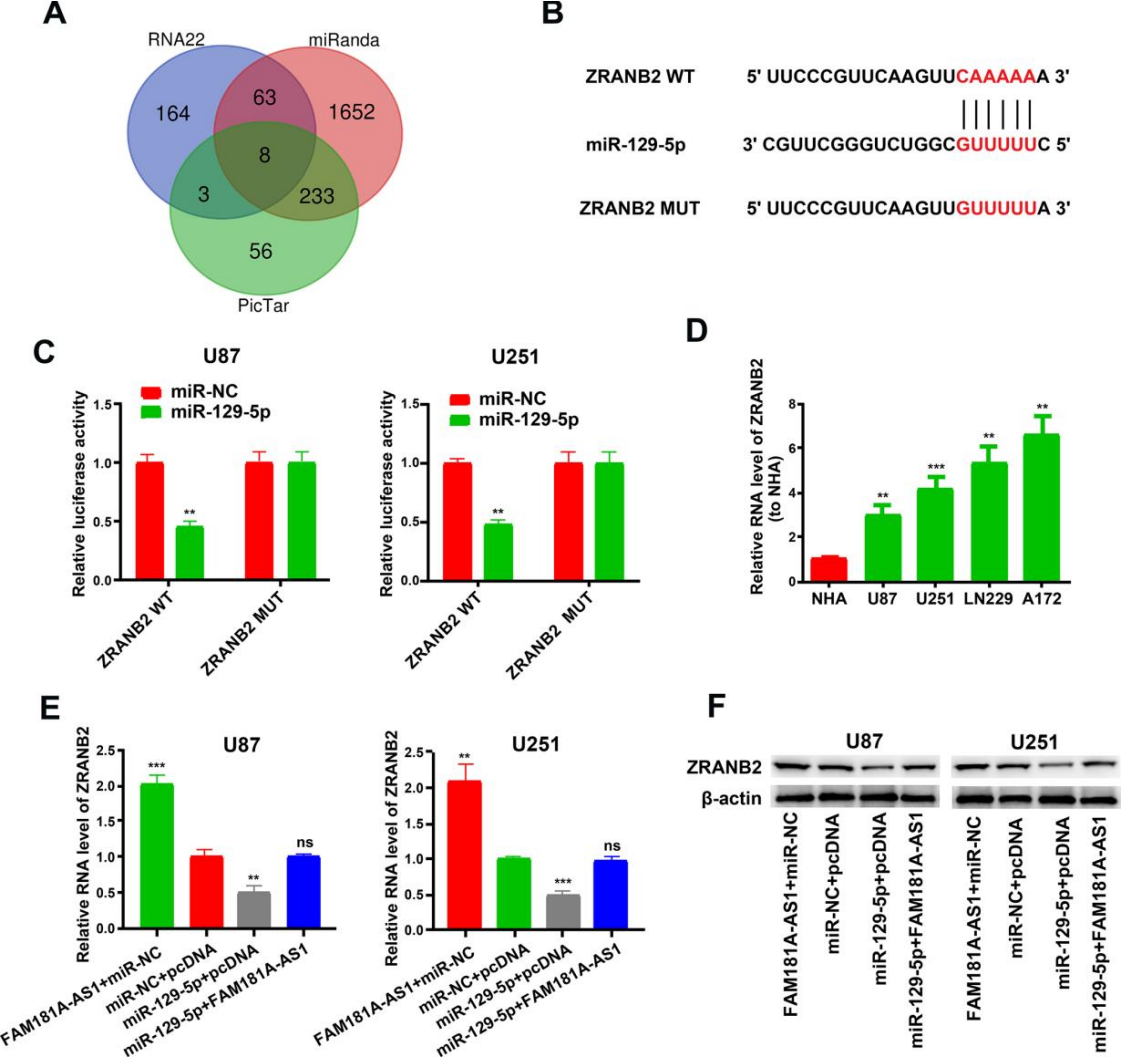
***ZRANB2* is a downstream target gene of miR-129-5p.** (**A**) RNA22, miRanda and PicTar analyses results show identification of 8 potential miR-129-5p target genes, namely, *ZNF646, RBPJ, CAMTA1, FBXW7, ZRANB2, L1CAM, PDS5A* and *ZFHX3*. (**B**) Diagrammatic representation shows miR-129-5p binding sites in the WT and MUT 3'UTR of *ZRANB2*. (**C**) Dual luciferase reporter assay results show relative luciferase activity in U87 and U251 cell lines transfected with miR-129-5p mimic and plasmid vectors carrying WT or MUT 3′UTR of *ZRANB2*. (**D**) QRT-PCR results show *ZRANB2* mRNA levels in glioma cell lines and the control NHA cell line. (**E**, **F**) QRT-PCR and Western blot analyses show *ZRANB2* mRNA and protein levels, respectively, in *FAM181A-AS1-*overexpressing U87 and U251 cell lines transfected with miR-NC or miR-129-5p mimic. Note: **P<0.01, ***P<0.001; ns, not statistically significant; data are represented as means ± SD.

Previous studies show that miR-129-5p impacts cellular pathways by modulating target gene expression including IGF-1R, HMGB1, PBX3 and so on [21–23]. We analyzed the RNA22, miRanda and PicTar databases and found 8 potential miR-129-5p target genes, namely, *ZNF646, RBPJ, CAMTA1, FBXW7, ZRANB2, L1CAM, PDS5A,* and *ZFHX3* (Figure 7A). We chose *ZRANB2* for further analysis because it is previously shown to be associated with glioma [24]. Figure 7B shows the miR-129-5p binding sites in the wild-type or mutant 3’UTR of *ZRANB2*. Dual luciferase reporter assay results showed that luciferase activity was significantly lower in U87 and U251 cells transfected with miR-129-5p mimic plus plasmid vector containing WT-3’UTR of *ZRANB*2 compared to those transfected with miR-129-5p mimic plus plasmid vector containing MUT-3’UTR of *ZRANB2* (Figure 7C). Furthermore, *ZRANB2* mRNA levels were significantly higher in the four glioma cell lines compared with the NHA cell line (Figure 7D). QRT-PCR and western blot analysis showed that ZRANB2 mRNA and protein levels were significantly decreased when U87 and U251 cells were transfected with the miR-129-5p mimic, but these effects were reversed in U87 and U251 cells overexpressing *FAM181A-AS1* (Figure 7E–7F).

### *ZRANB2* overexpression promotes proliferation of *FAM181A-AS1* downregulating glioma cells

**Figure 8:**
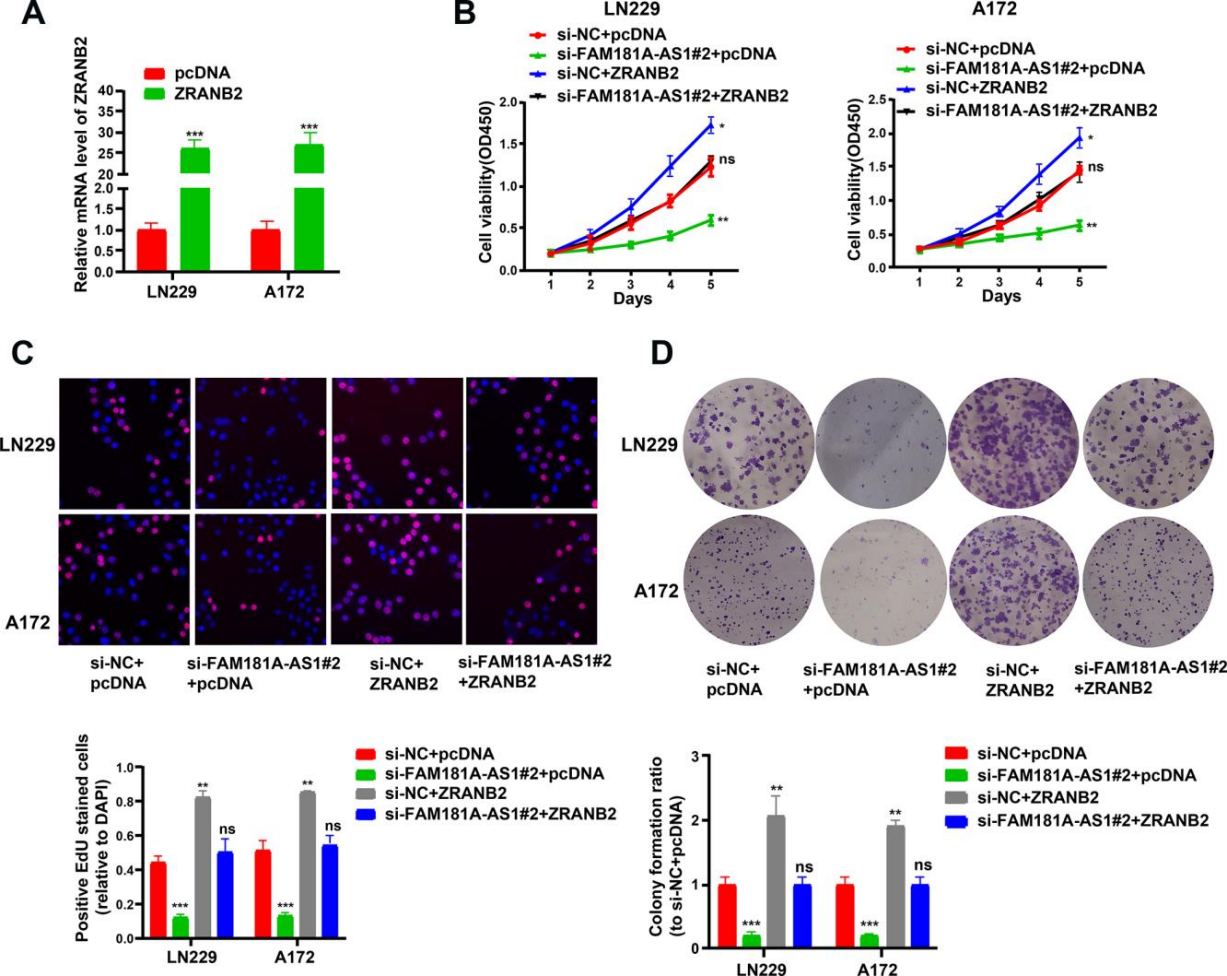
***ZRANB2* overexpression promotes proliferation of *FAM181A-AS1* downregulating glioma cells.** (**A**) Transfection efficiency of *ZRANB2*-expression plasmid in LN229 and A172 cell lines is examined through qRT-PCR. (**B**, **C**) CCK-8 and EdU analyses of the cell viability after transfection with *FAM181A-AS1* siRNA and (or) *ZRANB2*-expression plasmid in LN229 and A172 cell lines. (**D**) Colony formation analysis of the cell colony abilities after transfection with *FAM181A-AS1* siRNA plasmid and (or) *ZRANB2*-expression plasmid in LN229 and A172 cell lines. **P<0.01, ***P<0.001, ns: no statistical significance, data represent the mean ± SD.

Next, we analyzed the role of ZRANB2 in gliomagenesis by performing plasmid overexpression in glioma cell lines and assaying their cell growth and viability. QRT-PCR analysis showed that ZRANB2 mRNA levels were significantly upregulated in ZRANB2-expression plasmid-transfected LN229 and A172 cells compared to the corresponding controls (Figure 8A). ZRANB2 overexpression in FAM181A-AS1-downregulating LN229 and A172 cells showed significantly increased proliferation and colony formation compared to FAM181A-AS1 downregulating LN229 and A172 cells (Figure 8B–8D).

## DISCUSSION

Recent advances in transcriptomics have highlighted the significant functions of several lncRNAs in tumorigenesis and cancer therapy. *LINC00174* knockdown improves the response of human glioma cells to temozolomide by modulating the miR-138-5p/SOX9 axis [25]. LncRNA *CCAT1* enhances the growth of gliomas by sponging miR-181b [26]. Besides, lncRNA *UCA1* regulates glioma growth and metastasis via miR- 182-dependent regulation of iASPP protein [27]. These reports highlight the pivotal role of lncRNAs in regulating gliomagenesis. In this study, we analyzed the role of lncRNA *FAM181A-AS1* in gliomagenesis. The analysis of RNA-seq data in the TCGA and GTEx databases showed that *FAM181A-AS1* expression is significantly increased in glioma tissues compared to normal brain tissues. Moreover, CGGA database analysis showed that high *FAM181A-AS1* expression correlates with worse survival rates of glioma patients. Furthermore, *FAM181A-AS1* overexpression significantly increased proliferation and colony formation of glioma cell lines, whereas, these effects were reversed in *FAM181A-AS1* knockdown glioma cell lines.

LncRNAs modulate cellular growth and survival by acting as ceRNAs that sponge miRNAs [28, 29]. We demonstrate an inverse relationship between *FAM181A-AS1* and miR-129-5p expression in glioma samples. Previous studies show that miR-129-5p inhibits growth and progression of gastric cancer [30], breast cancer [31], colon cancer [32] and chondrosarcoma [33]. In our study, we demonstrate that miR-129-5p overexpression decreases proliferation of *FAM181A-AS1*-overexpressing glioma cells. Besides, miR-129-5p expression correlates with the clinical stage and overall survival of glioma patients. Overall, our data suggests that miR-129-5p plays an inhibitory role in gliomas, whereas, *FAM181A-AS1* promotes growth of glioma cells by sponging miR-129-5p.

Our study also confirms that *ZRANB2* expression is regulated by miR-129-5p in glioma cells. *ZRANB2* is an RNA-binding protein that was initially discovered in the rat juxtaglomerular cells [34]. ZRANB2 binds to Smad and suppresses the bone morphogenetic protein (BMP) signaling pathway in HEK293T cells [35]. Besides, *ZRANB2* is highly expressed in the serous papillary carcinomas of the ovaries [34]. Li et al. showed that glioma cells and tissues overexpress *ZRANB2* and its downregulation reduces proliferation, migration, invasion and vasculogenic mimicry of glioma cells [24]. Therefore, our results demonstrate that lncRNA *FAM181A-AS1* promotes growth of gliomas by increasing ZRANB2 expression via sponging of miR-129-5p.

This study has several limitations. Firstly, we lack glioma tissue samples. Hence, in the future, a larger patient sample size is required to confirm the clinical value of *FAM181A-AS1* in glioma patients. Secondly, we did not analyze other *FAM181A-*AS1-related target genes and miRNAs.

In conclusion, our study demonstrates that lncRNA *FAM181A-AS1* promotes growth and survival of glioma cells by enhancing *ZRANB2* expression via sponging of miR-129-5p. Our study suggests that lncRNA *FAM181A-AS1* is a potential prognostic biomarker and therapeutic target for glioma patients.

## MATERIALS AND METHODS

### Identification *FAM181A-*AS1-specific miRNAs and miR-129-5p target genes

We screened the microRNA.org database (http://www.microrna.org) to identify for *FAM181A-AS1*-binding microRNAs and miR-129-5p target genes.

### Cell culture

We purchased normal human astrocyte (NHA) cell line and four glioma cell lines (LN229, A172, U87 and U251) from the Institute of Biochemistry and Cell Biology of the Chinese Academy of Sciences (Shanghai, China). They were cultured in DMEM (GIBCO-BRL) medium with 10% FBS, 100 mg/mL streptomycin and 100 U/mL penicillin in a humidified incubator at 37° C and 5% CO_2_.

### Quantitative real-time PCR (qRT-PCR)

Total RNA was extracted using TRIzol (Beyotime, Nantong, China) according to manufacturer’s instructions. Equal amounts of total RNA were reverse transcribed using M-MLV, and the cDNA was amplified and quantified using PrimeScript™ Reverse Transcription reagent kit (Takara, Dalian, China) according to the manufacturer’s instructions. We used U6 and GAPDH as internal controls to quantify expression of miR-129-5p and *FAM181A-AS1*, respectively, using the 2^-ΔΔCt^ method. All experiments were repeated thrice. The primers used in this study are:

*FAM181A-AS1* forward: 5'-GAGACCCAGTAAAGCCCACT-3';

*FAM181A-AS1* reverse: 5'-TTCAGGGCCTGCATAGAACA-3';

MiR-129-5p forward: 5′-ACCCAGTGCGATTTGTCA-3';

MiR-129-5p reverse: 5'-ACTGTACTGGAAGATGGACC-3';

GADPH forward: 5'-AGTAGAGGCAGGGATGATG-3';

GAPDH reverse: 5'-TGGTATCGTGGAAGGACTC-3';

U6 forward: 5'-GGTCGGGCAGGAAAGAGGGC-3';

U6 reverse: 5'-CTAATCTTCTCTGTATCGTTCC-3';

### Western blotting

Total protein lysates were prepared by incubating cells with RIPA buffer (Beyotime Institute of Biotechnology, China) for 3 min followed by centrifugation at 17,000 g for 45 mins at 4° C. Then, the protein concentrations were determined using a BCA protein assay kit (Beyotime, Nantong, China). Equal amounts of total protein lysates were separated on a 10% SDS-PAGE and then transferred onto PVDF membranes. Then, the PVDF membranes were incubated with 5% skim milk in Tris-buffered saline with Tween (TBST) for 2 h at room temperature. The membranes were incubated with primary antibodies against zinc-finger RAN-binding domain containing protein 2 (*ZRANB2*, 1:500; Proteintech, Rosemont, IL, USA), Cyclin D1 (ab16663, 1:100; Abcam, Shanghai, China), Bcl-2 (ab182858, 1:1000; Abcam, Shanghai, China) and PCNA (ab29, 1:500; Abcam, Shanghai, China) overnight at 4° C. Subsequently, after rinsing the membranes thrice with TBST, the membranes were incubated with HRP-conjugated secondary antibodies (1:1,000; Beyotime, Nantong, China) for 2 h at room temperature. Then, the blots were developed using enhanced chemiluminescence (ECL) Kit from Beyotime Institute of Biotechnology (China) and visualized using the ChemImager 5500 V2.03 software from Alpha Innotech (San Leandro, CA). The integrated density values (IDVs) of the protein bands were quantified using GAPDH as the internal control.

### Dual luciferase reporter assay

We seeded U87 and U251 cells in 24-well plates for 24 h. Thereafter, we co-transfected the cells with 6 ng of pmirGLO vectors carrying wild-type (WT) or mutant (MUT) *FAM181A-AS1* or WT or MUT 3’UTR of *ZRANB2* and 100 nmol/L of miR-129-5p or miR-NC. Similarly, we co-transfected the cells with 6 ng of pmirGLO vectors carrying wild-type (WT) or mutant (MUT) *miR-129-5p* and pcDNA3.1-*FAM181A-AS1* plasmids or pcDNA3.1 negative control. After 48 h, we used the Dual luciferase Reporter System (Promega, Madison, WI, USA) to analyze the firefly and Renilla luciferase activities according to manufacturer’s instructions.

### Cell transfections

We purchased pcDNA3.1, pcDNA3.1-*FAM181A-AS1* plasmids and pcDNA3.1-*ZRANB2* plasmids, si-NC and si-*FAM181A-AS1*, as well as miR-NC and miR-129-5p mimic from Genepharma (Shanghai, China). For transfections, we seeded LN229, A172, U87 and U251 cell lines in six-well plates for 24 h, and performed transfections with plasmids, siRNAs, or miRNA mimics at 50-70% confluence using Lipofectamine 2000 (Invitrogen, Shanghai, China) according to manufacturer’s instructions.

### Cell proliferation assays

For the CCK-8 assay, we seeded 1-2×10^3^cells/well in 96-well plates overnight at 37° C and 5% CO_2_. Then, we used the CCK-8 kit (Dojindo Laboratories, Shanghai, China) to determine cell proliferation at different time points. Briefly, the primary medium in the wells was replaced with 100 μL of complete medium with 10 μL of CCK-8 reagent, and the cells were incubated for 2 h. Then, the absorbance was determined at 450 nm using a plate reader.

Cells seeded in 96-well plates were labeled with 50 μM medium containing 5-ethynyl-2’-deoxyuridine (EdU; Ribobio Co., Ltd., Guangzhou, China) for 2 h, fixed with 4% paraformaldehyde and 0.5% Triton X-100 and incubated with anti-EdU working solution according to the manufacturer’s instructions. Cell nuclei were dyed with DAPI (Beyotime, Nantong, China). A total of five randomly selected fields of view in each well were captured using fluorescence microscopy (x200 magnification) to calculate EdU-positive cells. The experiments were performed in triplicate.

### Colony formation assays

For the colony formation assays, 2×10^3^ cells were seeded in 12-well plates and incubated for 1 week at 37° C and 5% CO_2_. Then, the colonies were stained using a solution containing 0.1% crystal violet and 20% methanol. The colonies in each well were counted under a light microscope.

### Measurement of apoptosis

Apoptotic cells were detected by using Annexin V-FITC/PI Apoptosis Detection Kit (Beyotime, China). After transfection, the cells were washed with PBS for three times and stained with 5 μL Annexin V-FITC for 5 min in the dark at room temperature, followed by being added with 10 μL Propidium Iodide (PI) for 15 min. Next, the samples were detected with BeamCyte (China) and analyzed with CytoSYS 1.0 software.

### Cell cycle analysis

Cell Cycle and Apoptosis Kit (Beyotime, China) was utilized to detect cell cycle. Briefly, cells were washed with pre-cooled PBS and fixed with 70% ethanol for 24 h overnight. After that, 0.2% Triton X-100 was added into the cell solution, followed by re-suspended with 100 μg/mL RAase A for 30 min. Next, 10 μL PI solution was added for staining at 37° C in the dark. BeamCyte (China) was used to analyze the results.

### RNA pull-down assay

Human glioma cells were lysed with cell lysis buffer (RNA pull-down kit, gzscbio, Guangzhou, China). The biotinylated *FAM181A-AS1* or negative control (GenePharma, Shanghai, China) was incubated with Dynabeads M-280 Streptavidin (Invitrogen, Shanghai, China) to generate probe-coated beads. The lysates were incubated with the probe-coated beads at 4° C overnight. Then, the RNA complexes bound to these beads were eluted with Wash Buffer (RNA pull-down kit, gzscbio, Guangzhou, China). Finally, the miR-129-5p levels in the pulled down RNA were quantified using qRT-PCR.

### RNA immunoprecipitation (RIP)

We performed the RIP assay using the Magna RIP RNA-binding protein immunoprecipitation kit (Millipore Corp., Billerica, MA, USA). We used rabbit polyclonal anti-Ago2 antibody (ab5072, Abcam) to pull down the RNA. Anti-IgG antibodies were used as negative control. Then, *FAM181A-*AS1 and miR-129-5p levels in the pulled down RNA was quantified using qRT-PCR.

### Statistical analysis

Statistical analyses were performed using SPSS 20.0 (IBM; Chicago, IL, USA) and GraphPad Prism (GraphPad software, San Diego, CA, USA) software. The differences between groups with normally distributed data with equal variance were evaluated using 2-tailed Student t test (2-group comparisons) or ANOVA followed by the post-hoc Bonferroni test (multigroup comparisons) as appropriate. The differences between groups with non-normally distributed data or unequal variance were evaluated using nonparametric Mann-Whitney U test (2-group comparisons) or the Kruskal-Wallis test followed by the post-hoc Bonferroni test (multigroup comparisons). Pearson's correlation coefficient analysis was used to determine the association between *FAM181A-AS1* and other miRNAs. Kaplan-Meier survival curve analysis and log-rank test was used to analyze prognostic significance. Receiver operating characteristic (ROC) curves analysis was performed to investigate the association between glioma and FAM181A-AS1/miR-129-5p expression levels. The data are presented as means ± SD. *P*<0.05 was considered statistically significant.

## Supplementary Material

Supplementary Figures
