# Supplementary material for: LncRNA FAM181A-AS1 promotes gliomagenesis by sponging miR-129-5p and upregulating ZRANB2
Source: Aging (Albany NY). 2020 Oct 20;12(20):20069–84. doi: 10.18632/aging.103391 (PMC7655169; doi:10.18632/aging.103391)
Supplement: Supplementary Figures [file aging-12-103391-s001..pdf]

SUPPLEMENTARY FIGURES

Supplementary Figures

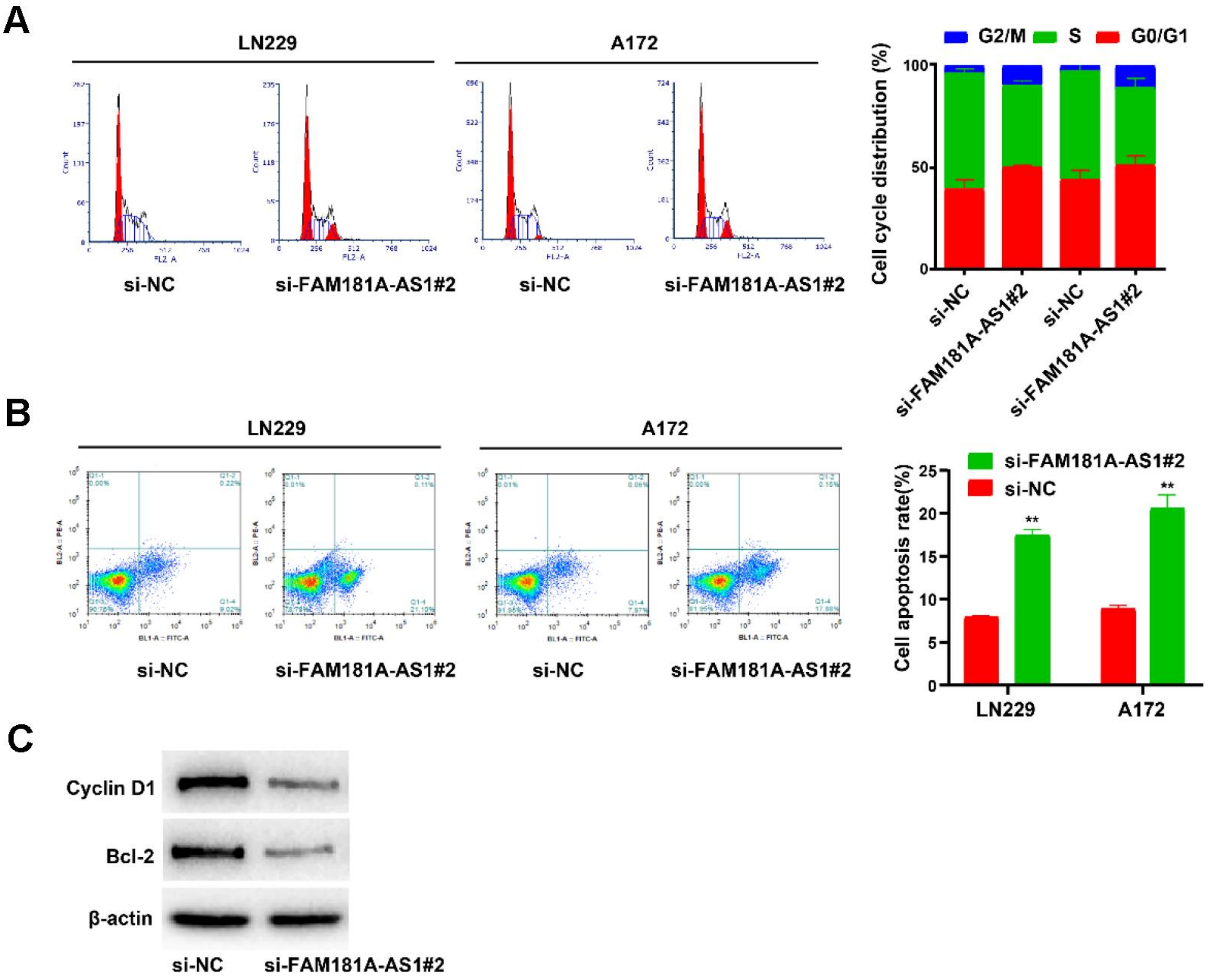

Supplementary Figure 1. *FAM181A-AS1* downregulation inhibits cell cycle and promotes cell apoptosis. (**A**) Cell cycle was detected. Cytometry showed the cells were arrested in G0/G1 phase after treated with *FAM181A-AS1* siRNA in LN229 and A172 cells. (**B**) Cell apoptosis was detected. Cytometry showed the downregulation of *FAM181A-AS1* promoted the apoptosis of LN229 and A172 cells. (**C**) Western blot showing that knockdown of *FAM181A-AS1* downregulated the expression of Cyclin D1 and Bcl-2. $^{**}P<0.01$, data represent the mean ± SD.

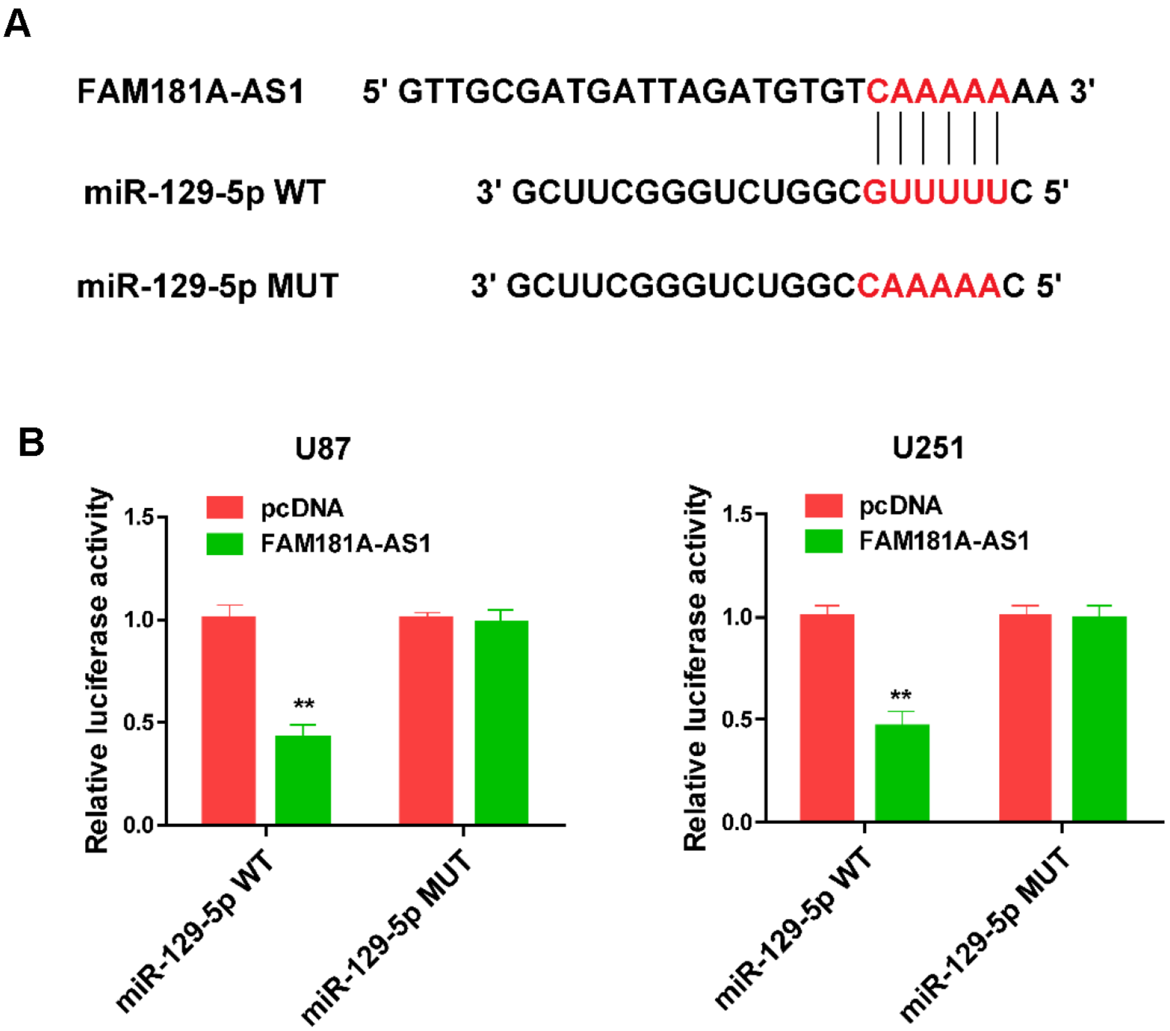

Supplementary Figure 2. The effect of *FAM181A-AS1* overexpression on the luciferase activity of miR-129-5p WT or MUT. (**A**) Images of the linking sites between *FAM181A-AS1* and miR-129-5p WT/MUT. (**B**) Assessment of the luciferase activity of miR-129-5p WT/MUT subsequent to transfection of U87 and U251 cell lines with *FAM181A-AS1* overexpression plasmid. **P<0.01, data represent the mean ± SD.
